# Fat Body Metabolome Revealed Glutamine Metabolism Pathway Involved in Prepupal *Apis mellifera* Responding to Cold Stress

**DOI:** 10.3390/insects16010037

**Published:** 2025-01-02

**Authors:** Xinjian Xu, Mingjie Cao, Chenyu Zhu, Lingqing Mo, Huajiao Huang, Jiaying Xie, Bingfeng Zhou, Shujing Zhou, Xiangjie Zhu

**Affiliations:** 1 College of Bee Science and Biomedicine, Fujian Agriculture and Forestry University, Fuzhou 350002, China; xuxinjian1982@126.com (X.X.); jack-2001@live.cn (M.C.); zcy1775880093@163.com (C.Z.); 3205602088@stu.fafu.edu.cn (L.M.); 3215602058@stu.fafu.edu.cn (H.H.); 3225602071@stu.fafu.edu.cn (J.X.); bingfengfz@126.com (B.Z.); 2 Honeybee Research Institute, Fujian Agriculture and Forestry University, Fuzhou 350002, China

**Keywords:** honeybees, fat body, metabolomics, oxidative stress, cold stress

## Abstract

Thermal condition affects the development and growth of ectotherms. The stenothermic honeybee brood, particularly the prepupae, are sensitive to low rearing temperature. The fat body plays important roles in energy reserve and metabolism during the honeybee brood development. To date, the fat body metabolic changes in prepupae responding to cold stress have not been completely understood. In this study, the ultra-performance liquid chromatography coupled with tandem mass spectrometry (UPLC-MS/MS)-based non-target metabolome was analyzed between the cold-treated (CT, 20 °C, 36 h) and control (CK, 35 °C) fat body in prepupal honeybees. The fat body metabolomic data showed that the levels of 1860 and 254 metabolites were significantly increased and decreased, respectively, in cold-stressed prepupae. These altered metabolites, glutamine, glutamic acid, pyroglutamic acid, and oxidized glutathione, were significantly enriched into glutamine metabolism and glutathione metabolism pathways. Furthermore, the expression levels of glutamine metabolism-related genes, *glutaminase* (*GLS*), *glutamate dehydrogenase* (*GDH*), and *gamma-glutamyl transferase* (*GGT-1* and *GGT-7*), were significantly decreased in cold-exposed prepupae compared with the control groups. Meanwhile, the oxidized glutathione (GSSG), but not the reduced glutathione (GSH) content, was increased in the cold-exposed group compared with controls. Collectively, our data revealed the fat body metabolomic changes in larva-to-pupa transition when exposed to cold stress. Our data provided new insights into stenothermic honeybee sensitivity to cold, characterized by perturbation of glutamine metabolism and oxidative stress.

## Simple Summary

Honeybees are holometabolous insects which complete the metamorphosis process by relying on stable hive temperatures regulated by the colony. Our previous study found that prepupae of honeybees are sensitive to low-temperature stress, which led to delayed or failed pupation process. Prepupae mobilized and utilized energy and resources stored in larval fat bodies (nucleotides, fatty acids, amino acids, etc.) to construct new pupal tissues and organs. We performed non-target metabolomics to identify the key differential metabolites, and the genes in associated metabolic pathways to analyze the low-temperature stress effect on metamorphosis development. The findings indicate that cold stress disrupts the metabolism of the glutamine and glutamate metabolism and glutathione metabolism, thereby impairing honeybee development.

## 1. Introduction

The thermal conditions significantly influence the development and growth of ectotherms [1,2]. Honeybee broods exhibit stenothermy, being exclusively adapted to a narrow temperature range, from 33 °C to 36 °C [3,4,5], with 35 °C being the optimal temperature [6]. Deviations in rearing temperature from this optimal range can have detrimental effects on the later stages of honeybee brood development [7,8]. Consequently, adult worker honeybees that develop under suboptimal conditions are less effective in meeting the workforce demands of the colony. This leads to decreased foraging, heating, and nursing performances, creating a detrimental cycle that culminates in colony collapse [9].

Exposure to cold stress during early life can impede development and lead to enduring implications in honeybees. Research has demonstrated a 40% reduction in survival among honeybee larvae exposed to 25 °C temperatures [10]. Cold stress during the larval stage reduced metabolic rates, prolonged development, increased pupal mortality, and heightened susceptibility to pesticides [11]. Exposure of pupal honeybees to suboptimal rearing temperatures, specifically 20 °C for 12 h, resulted in a 52% failure rate in completing the molting process in subsequent stages [8]. This exposure also led to neurological deficits, impaired learning, and memory issues in adult honeybees [12,13,14]. The honeybee prepupae are particularly susceptible to cold-induced harm [8], although the precise mechanism behind this vulnerability remains unknown.

The fat body plays a vital role as the primary energy source during honeybee metamorphosis. However, the metabolic changes in this organ under cold stress are still not well understood. As honeybees progress from larval instar to pupae, the fat body undergoes dissociation, apoptosis, and remodeling [15,16]. This process enables the fat body to utilize lipids, carbohydrates, and proteins to generate, store, and mobilize energy during pupal development, supporting overall growth [17]. The metabolic processes of fat body tissue may be influenced by cold stress, potentially impacting the crucial tissue remodeling during metamorphosis. Exposure to cold stress alters the morphology of the fat body, with freezing stress below 0 °C causing lipid droplet fusion, disruption of α-tubulin organization, and noticeable F-actin aggregation in malt fly larvae [18]. Additionally, cold stress can induce metabolic changes in fat body tissue. Subsequent to cold stress, a notable elevation in the total protein content was observed in the fat body of adult Madagascar cockroaches (*Gromphadorhina coquereliana*), while lipid and glycogen levels remained unchanged [19]. However, under mild cold conditions, there were no significant alterations in carbohydrate, lipid, and protein levels, although a noticeable shift in mitochondrial respiration activity was observed [20]. These findings suggest that fat body tissue may enhance respiratory metabolism at low temperatures, yet the increased energy consumption during the developmental phase could impact later developmental stages, given the critical role of this energy budget in the developmental process of holometabolous insects. In light of the above, it is imperative to conduct a thorough investigation into the metabolic alterations of the prepupal fat body in bees subjected to low-temperature stress.

In our previous study by Wang et al. (2016) [8], it was observed that prepupal honeybees exhibit a higher sensitivity to low-temperature stress compared to other stages of honeybee brood development. The underlying mechanism of this phenomenon remains largely unexplored. Our hypothesis posits that cold stress may interfere with the normal metabolic processes during honeybee development under stress conditions. To investigate this, a comparative non-targeted LC-MS/MS metabolomic analysis was conducted on cold-stressed and control fat body samples from prepupae. The aim was to identify key metabolites and pathways that play a role in modulating developmental damage and responses to cold exposure. This research aims to establish a foundation for the controlled regulation of developmental temperatures to enhance honeybee development and overall health.

## 2. Materials and Methods

### 2.1. Honeybee Capped Brood and Cold Treatment

The honeybee *Apis mellifera* colonies were reared in Fuzhou, China, according to standard beekeeping techniques. All colonies had adequate honey and bee bread store, as well as nonvisible symptoms of any known brood disease. Each colony consisted of a young egg-laying queen and a working population of 10 standard frames of worker bees in Langstroth hives. For the purpose of obtaining cohorts of honeybee brood at identical developmental stages, the queen was restricted within a mesh cage to lay eggs on a new comb over a period of 12 h. On the 9th day, the larvae were monitored until capped. The comb patch with the newly capped brood sealed within 4 h was cut down and transferred to the incubators (35 °C ± 0.2 °C, RH 75% ± 5%, ALISN, Shanghai, China).

Preliminary experiments revealed a significant delay in the development of capped honeybee broods when subjected to cold treatment at 20 °C, resulting in the failure of prepupae to transition into pupae. Prepupae subjected to 18 h and 36 h low-temperature treatments remained in their prepupal stage, while those in the control group successfully underwent pupation [8]. Based on these findings, the following experimental treatments were designed. The newly capped brood was incubated at 35 °C ± 0.2 °C (RH 75% ± 5%) for 3 d until reaching the prepupal stage. Subsequently, the prepupae within comb patches were subjected to either 20 °C ± 0.2 °C (RH 75% ± 5%) for 18 or 36 h (cold-treated, CT18, and CT36) or 35 °C ± 0.2 °C for 18 or 36 h (controls, CK18, and CK36) before manual uncapping and collection of the brood. Ten broods per each replicate were uncapped and immediately immersed into liquid nitrogen and stored at -80 °C for further analysis. In total, 6 replicates of CT36 vs. CK36 samples were subjected to metabolome analysis, and 3 replicates of CT18 vs. CK18 and CT36 vs. CK36 samples were used for gene expression or biochemical analysis.

### 2.2. Fat Body Metabolite Identification

A comparative metabolome analysis was conducted on CK36 and CT36 brood samples. Individual brood specimens were dissected, and their fat body tissues were collected in Eppendorf tubes. Six biological replicates per group were homogenized through ultrasonication and then stored at −20 °C for 2 h to precipitate. After centrifugation at 25,000× *g* for 15 min at 4 °C, the supernatants were transferred to new tubes for drying in a centrifugal evaporator. Subsequently, 600 µL of a 10% methanol solution was added to the samples, followed by centrifugation at 25,000× *g* for 15 min at 4 °C. Then, 5 µL of each sample was injected, and quality control (QC) samples were prepared by mixing 50 µL from each sample.

Metabolite separation was performed using an ultraperformance liquid chromatography system (2777C, Waters, Milford, MA., USA) coupled with an ACQUITY HSS T3 column (100 × 2.1 mm, 1.8 µm, Waters, USA). The column temperature was maintained at 50 °C, with a flow rate of 0.4 mL per minute. The mobile phase consisted of solvent A (water + 0.1% formic acid) and solvent B (methanol + 0.1% formic acid). Metabolites were eluted using a gradient of 0–2 min (100% phase A), 2–11 min (0% to 100% B), 11–13 min (100% B), and 13–15 min (0% to 100% A). Detection of small molecules eluted from the column was carried out using the high-resolution tandem mass spectrometer Xevo G2 XS QTOF (Waters, USA) in both positive- and negative-ion modes. Capillary voltages were set at 3 kV (+) and 2 kV (–), with a sampling cone voltage of 40 V in both modes. Data collection was performed in Centroid MSE mode, with a first-stage scan range of time-of-flight mass from 50 to 1200 Da and a scan time of 0.2 s. Tandem mass spectrometry (MS/MS) detection involved fragmenting parent ions using 20–40 eV, with a scan time of 0.2 s. Mass accuracy calibration was conducted by performing the LE signal every 3 s during data collection.

### 2.3. Metabolite Data Process and Analysis

The raw metabolomics data obtained from the mass spectrometer were subjected to analysis using Progenesis QI 2.2 (Waters, MA, USA), including peak alignment, peak picking, normalization, deconvolution, and peak identification. Then, the data were processed with the metabolomics analysis R package metaX (BGI, Shenzhen, China) for further analysis. First, the low-weight ions with a relative standard deviation > 30% were filtered and removed to ensure the metabolic quality. In addition, the data were corrected by QC-RLSC (QC-based robust LOESS signal correction) method.

Next, fold change (FC) detection was performed in the univariate analysis, with the quantification of each metabolite. Simultaneously, *t*-test analysis was used to calculate the *p*-value. In the multivariate analysis, principal component analysis (PCA) was performed for general clustering between the groups, as the influence on the intensity and explanatory capacity of each ion was calculated by VIP (variable importance of projection), and components with VIP > 1 were considered to be potential compounds contributing significantly to the differences between groups. Based on the results of QC sample detections, the RSD threshold was set below 30% for error reduction, which was reflected in the FC of metabolites.

Metabolite annotation was performed using the Human Metabolome Database (HMDB), Kyoto Encyclopedia of Genes and Genomes (KEGG), and LipidMaps. The differential metabolites (DMs) between the CT vs. CK were screened by the criteria (VIP > 1, *p* < 0.05, FC < 0.7 or FC > 1.5). The pathway associated with the DMs was enriched in an R package ClusterProfiler on the Biomarker online platform (http://www.biomarker.com.cn/technology-services/feibaxiang, accessed on 5 October 2023).

### 2.4. Measurement of Gene Expression Levels

To investigate the activity of glutamine metabolism pathway in fat body response to cold stress, we quantified the expression levels of genes of *glutaminase* (*GLS*), *glutamate dehydrogenase* (*GDH*), and *gamma-glutamyl transferase* (*GGT-1* and *GGT-7*) in prepupae exposed to 20 °C (CT18 vs. CK18 and CT36 vs. CK36, likewise in 2.1). The total RNA was extracted from 3 prepupal bee fat body (per replicate), and each 1000 ng total RNA sample was then reverse-transcribed to the first-strand cDNA following the manufacturer’s instructions. The real-time quantitative PCR (RT-qPCR) based on SYBR green fluorescence was performed using SYBR^®^ Premix Taq (Tiangen, Beijing, China). The reactions were run on an ABI Q5 System (ABI, San Francisco, CA., USA) using the following protocol: 40 s at 95 °C, followed by 40 cycles of 95 °C for 5 s and 60 °C for 30 s. Each RT-qPCR assay consisted of three biological replicates with three technical replicates. The housekeeping gene *β-actin* was used as the inner reference [21], and the primer sequences of four target genes are given in Table 1. The gene expression levels were analyzed using the 2^−ΔΔCt^ method [22].

### 2.5. Biochemical Assay

The concentrations of reduced glutathione (GSH) and oxidized glutathione (GSSG) were assessed in the fat body of honeybee prepupae exposed to cold treatment (CT18 vs. CK18; CT36 vs. CK36). Fat body tissue was dissected from each prepupal sample, processed as described in Section 2.4, and subsequently combined from three honeybees prior to being stored at −80 °C. Three biological replicates were assessed for each group, following the guidelines provided by the manufacturer (G0206W and G0207W, https://www.geruisi-bio.com/, accessed on 6 April 2023).

### 2.6. Data Analysis

The study utilized Student’s *t*-test to assess the differences in gene expression levels and biochemical indexes between the cold-treated groups and controls. Statistical significance was defined as *p* < 0.05 (*) and *p* < 0.01 (**).

## 3. Results

### 3.1. Data Assessment and Analysis of Significantly Differential Metabolites

**Figure 1:**
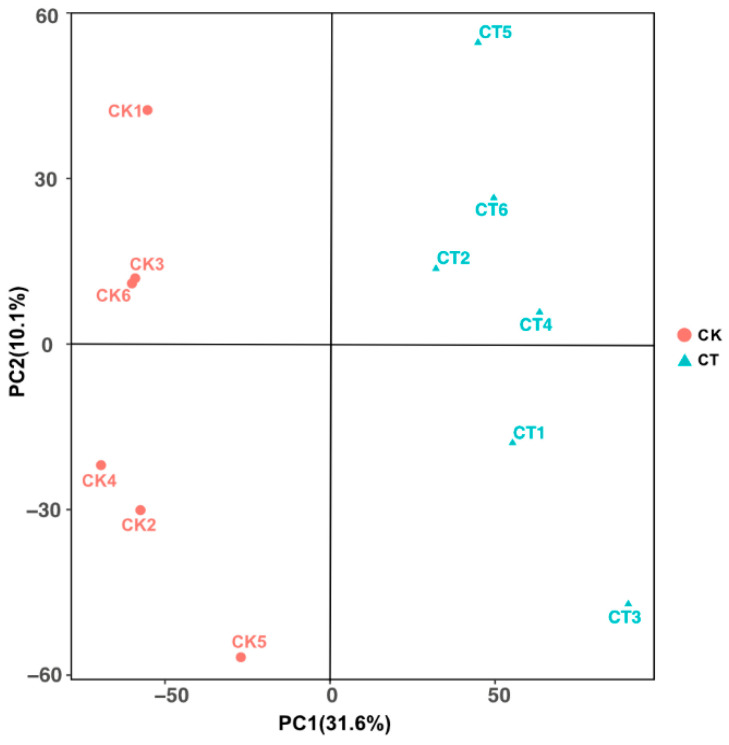
Principal component analysis (PCA) of metabolites obtained from fat body between the CT36 and CK36 (6 biological replicates). Principal components 1 and 2 account for 31.6% and 10.1% metabolite variation, respectively.

Principal component analysis (PCA) was conducted to assess the group differences between the cold-treated and control groups. Principal components 1 and 2 account for 31.6% and 10.1% metabolite variation, respectively. The results of clustering showed that CT36 replicates and CK36 replicates could be clearly separated (Figure 1) based on the metabolite profile. The data revealed that there was a significant change in fat body metabolome of prepupal *Apis mellifera* in response to cold exposure compared with the control group.

**Figure 2:**
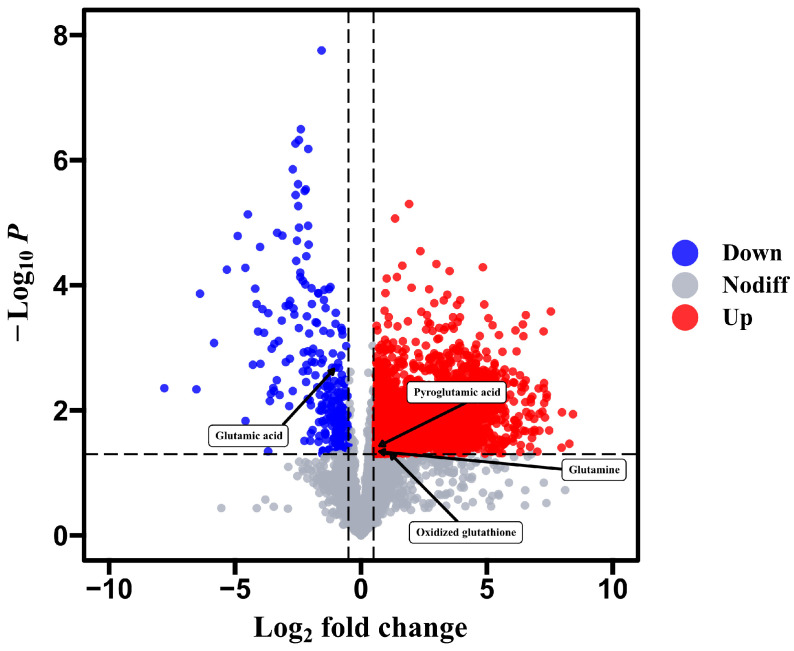
Volcano plot of different metabolites identified in prepupal honeybee fat body between the CT36 and CK36 groups, based on FC > 1.5 or FC < 0.7, *p* < 0.05. Each point represents a metabolite, and red dots and blue dots represent upregulated and downregulated metabolites. The *x*-axis represents the logarithm of metabolite fold change (FC) values, and the *y*-axis represents the *p*-value obtained by Student’s *t*-test.

To further screen the fat body metabolites in response to cold stress, a total of 2114 significantly differential ions out of 11879 ions showed significant changes between CT36 and CK36, with 1860 and 254 ions significantly increased and decreased, respectively (Figure 2), based on screening thresholds (FC > 1.5 or FC < 0.7, *p* < 0.05, Variable Importance in Project (VIP) > 1.0). A total of 572 ions were annotated in HMDB and KEGG database, and the DMs significantly differed between the CT36 and CK36.

### 3.2. Enrichment Analysis of Metabolites

To determine the potential metabolic pathways altered during cold stress, KEGG pathway enrichment analysis for CT36 vs. CK36 was performed based on the identified DMs. Only 71 DMs out of the annotated DMs were enriched into 20 KEGG pathways, and the top ten KEGG pathways are as follows: glutamine/glutamate metabolism (4 DMs); Glyoxylate and Dicarboxylate metabolism (3 DMs), Nitrogen metabolism (3 DMs), Glutamatergic synapse(3 DMs), GABAergic synapse (3 DMs); Proximal tubule bicarbonate reclamation (2 DMs), Nicotine addiction (3 DMs), Glutathione metabolism (4 DMs), Alanine, aspartate and glutamate metabolism (3 DMs), and Metabolism of xenobiotics by cytochrome P450 (2 DMs).

**Figure 3:**
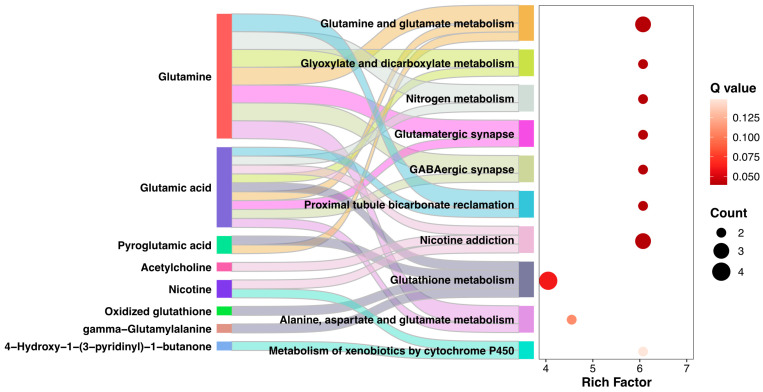
Top 10 KEGG pathways enriched by differential metabolites between CT and CK. The colored strip flow indicates each metabolite mapping to a KEGG pathway. The bubble size and color refer to the number of metabolites enriched and Q value for enrichment. The Rich Factor refers to the ratio of the number of metabolites enriched into the KEGG pathways to the number of annotated metabolites.

The altered metabolites, particularly the glutamine, glutamic acid, pyroglutamic acid, were shared and enriched into multi-KEGG pathways, suggested they play important roles and could be marker compounds in prepupal honeybees responding to cold stress (Figure 3).

### 3.3. Gene Expression Quantification

**Figure 4:**
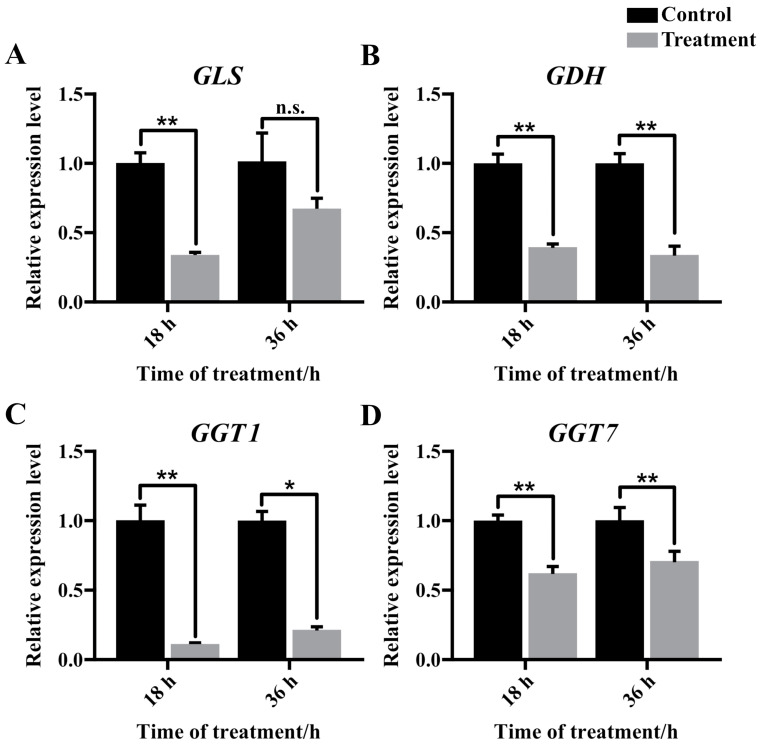
Relative expression levels of *GLS* (**A**), *GDH* (**B**), *GGT1* (**C**), and *GGT7* (**D**) in prepupae after 18 h and 36 h of exposure to cold vs. control. n.s. denotes not significantly different; * and ** denote *p* < 0.05 and *p* < 0.01, respectively.

The expression of *GLS* was significantly reduced in the 18 h cold-treated group (CT18) compared to the control group (CK18). Although there was a decreasing trend in *GLS* expression in the cold-treatment group at 36 h compared to the control group, statistical analysis did not show a significant difference (Figure 4A). *GDH* expression was significantly decreased after both 18 h and 36 h of cold treatment (CT18 and CT36) compared to the CK groups (CK18 and CK36) (Figure 4B). The expression of two GGT genes (*GGT1* and *GGT7*) was significantly downregulated following cold treatment in the cold-treated group compared to the control group (Figure 4C,D).

### 3.4. Glutathione Content in Cold Stressed Fat Body

**Figure 5:**
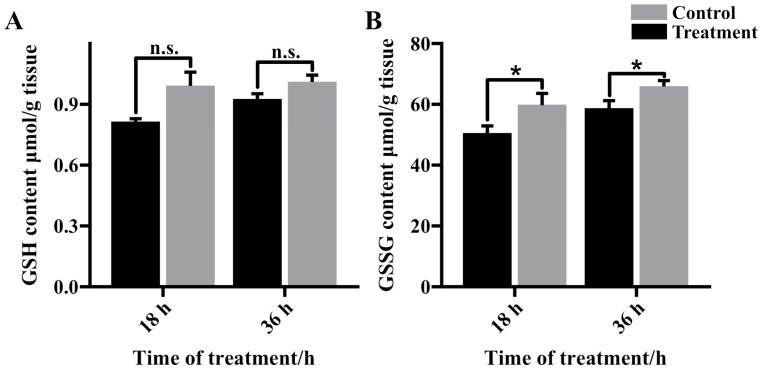
GSH (**A**) and GSSG (**B**) content in prepupae after 18 and 36 h of exposure to cold. n.s. denotes not significantly different; * denotes *p* < 0.05.

Following 18 h and 36 h cold treatments (CT18 and CT36) in prepupal fat body tissues, there was no significant alteration in reduced glutathione (GSH) content compared to the control (CK18 and CK36) (Figure 5A). In contrast, the levels of oxidized glutathione (GSSG) exhibited a notable increase after 18 and 36 h of cold stress compared to the controls (Figure 5B), aligning with the elevated GSSG content identified through metabolomic analysis.

## 4. Discussion

The precise regulation of honeybee brood-rearing temperature within a colony is essential for ensuring the completion of normal development processes. In subtropical regions, cold waves can lead to partial exposure of honeybee brood to low temperatures when adult bees fail to adequately warm the brood area in early spring. Previous studies have shown that capped honeybee broods are particularly vulnerable to suboptimal temperatures, especially during the prepupal stage [8]. In the metamorphic process, the larval fat body undergoes remodeling, a common phenomenon in many holometabolous insects. The fat body plays a crucial role in fueling normal growth and development by releasing and mobilizing resources, including lipids, carbohydrates, and proteins, as well as contributing to immune responses against the external environment [17]. The identification of 2114 differentially expressed metabolites in the fat body provides valuable insights into understanding the susceptibility of prepupal honeybees to cold stress. The top five significantly enriched pathways are glutamine and glutamate metabolism, glyoxylate and decarboxylate metabolism, nitrogen metabolism, glutamatergic synapse, and GABAergic synapse (Figure 3). Among these pathways, three differentially expressed metabolites (DMs) were identified: two upregulated DMs, glutamine and pyroglutamic acid; and one downregulated DM, glutamic acid. These findings suggest that cold stress during the honeybee prepupae stage may impact the transition between glutamic acid and glutamine in the fat body tissue. This process may be linked to energy metabolism and glutathione metabolism, potentially influencing the honeybee prepupal response to cold stress.

**Figure 6:**
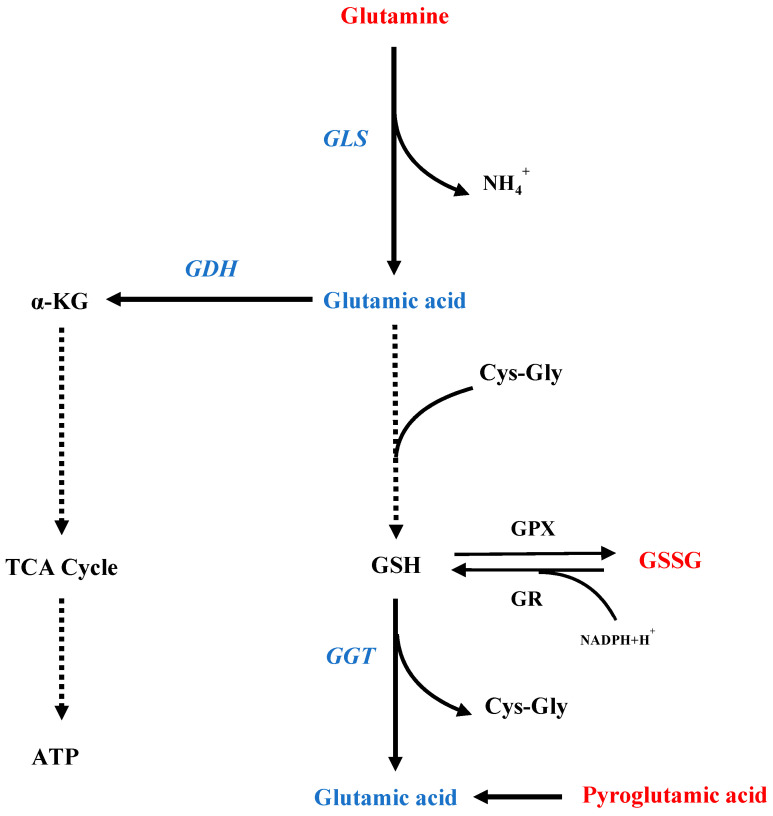
A schematic overview depicting a potential operational model of the glutamine metabolism pathway in honeybee prepupae post-cold stress. The red characters indicate upregulated metabolites and expression level of enzyme genes, while the blue ones indicate decreased metabolite and expression level of enzyme genes.

If the energy metabolism in fat body is in disorder, there is a high probability that the developmental process of the honeybee would be perturbed. A large body of data showed that glutamine metabolism pathway, coupled with Glutathione metabolism pathway, played important roles in the proper growth in cells and antioxidative response to various stress [23,24,25]. We observed that the glutamine and pyroglutamic acid were higher, while glutamic acid was lower in cold-exposure prepupae, possibly due to the low temperature decreased turnover of glutamine into glutamic acid in prepupae (Figure 6). The downregulation of gene *glutaminase* (*GLS*) in cold-exposed fat body supported this hypothesis (Figure 4). In addition, the decreased expression of gene *glutamate dehydrogenase* (*GDH*) implies lower downstream metabolites (such as α-ketoglutarate and ATP) and energy supply deficit for larva–pupa transition and antioxidation capacity under cold, thus requiring further investigation.

In comparison to the control group, stressed prepupae exhibited significantly higher levels of pyroglutamic acid and lower levels of glutamic acid. Previous studies have indicated that NADH-based glutaminase (GLS) plays a crucial role in converting glutamine to glutamic acid [26]. Our research revealed a significant decrease in the expression of mitochondrial *GLS*, as shown in Figure 4A, which corresponded with the reduced levels of glutamic acid in cold-stressed prepupae fat body. Glutamic acid can further generate α-ketoglutarate through the action of glutamate dehydrogenase (GDH), allowing glutamine to enter the tricarboxylic acid cycle as α-ketoglutarate and produce ATP via Succinyl CoA Synthetase [27]. Quantitative PCR assays confirmed the significant downregulation of both *GLS* and *GDH* expression in the prepupal fat body following cold exposure, as depicted in Figure 4A,B. Consequently, we hypothesize that the inhibition of *GLS* and *GDH* expression at low temperatures may impede the conversion of glutamine to glutamic acid in fat body cells, indicating a suppression of energy metabolism in prepupal fat bodies under cold-stress conditions.

Furthermore, an increase in the duration of low-temperature treatment correlates with elevated gene expression levels of *GLS* and *GGT*, while the expression of *GDH* shows a consistent decrease (Figure 4). This response in honeybee prepupae may indicate a strategy to maintain glutamic acid levels in the fat body tissue. Previous research has suggested that glutamic acid plays a crucial role in facilitating coordinated neuro-muscular activities required for successful ecdysis [28], as well as being essential for the proliferation of pupal adult tissue [29]. It is proposed that honeybee prepupae may regulate glutamic acid levels to support pupation resumption upon returning to optimal developmental temperatures.

Suboptimal temperatures can induce oxidative stress in the prepupal fat body of honeybees. In our study, metabolomics reveals four significantly different metabolites associated with the glutathione metabolic pathway, including oxidized glutathione (GSSG) (Figure 3), which showed a significant increase after 36 h of cold treatment (Figure 2). Biochemical assays confirmed the elevation of GSSG levels following 18 h and 36 h of cold exposure (Figure 5B). We also found that levels of reduced glutathione (GSH) remained unchanged after cold stress (Figure 5A). The results indicate a reduction in the GSH/GSSG ratio, a well-established marker of oxidative stress [30]. Furthermore, the glutamine and glutamate metabolic pathways are crucial for the synthesis of GSSG/GSH redox pairs within cells [31]. The conversion of glutamic acid and cysteine to gamma-glutamyl-cysteine, a pivotal step in GSH synthesis, was hindered by the significant downregulation of glutamic acid [32,33]. On the other hand, the absence of fluctuations in GSH levels post-cold treatment could be linked to the reduction in *gamma-glutamyl transferase* (*GGT*) expression observed after cold treatment (Figure 5A, B). This could be to decrease the degradation of GSH and prevent a reduction in the GSH pool [34,35]. In summary, the inhibition of glutathione synthesis has the potential to weaken the antioxidant defenses of fat body cells, ultimately disturbing the transition from larva to pupa.

## 5. Conclusions

In our study, we found that exposure to cold stress resulted in metabolic profile alterations in the fat bodies of honeybee prepupae. The decreased expression of *GLS* and *GDH*, along with reduced levels of glutamic acid, in cold-stressed prepupae indicated disruptions in fat body energy metabolism and oxidative balance during the honeybee larva-pupa transition in response to cold conditions. Moreover, the decreased levels of the *GGT* genes correlated with glutathione content in the fat body indicated that a decrease in GSH degradation in honeybee prepupae is a strategy in response to cold. This research offers novel insights into the impact of cold stress on brood development and overall health.

## Figures and Tables

**Table 1:** Gene primers for real-time PCR.

Gene Name	Gene ID	Forward Primer 5′-3′	Reverse Primer 5′-3′
*β* *-actin*	XM_623378	TGCCAACACTGTCCTTTCTG	AGAATTGACCCACCAATCCA
*GLS*	LOC408991	CGCCACTCGAGTCTATCCAC	ATTCATTCGCTTCGCGTTCG
*GDH*	LOC409253	GAGCAGTCGGCAGTGACAAG	GTCATCCTGGAACGTTTGCC
*GGT-1*	LOC551194	GCGAGAAATGCGCAAACTCA	CTAGCATACGCGGAACTGGT
*GGT-7*	LOC551194	TGCTGAAGGAGGTCCACAGA	CCACCACCGAGACCAGTTTT

## Data Availability

The original contributions presented in this study are included in the article. Further inquiries can be directed toward the corresponding author(s).
